# Melatonin and the Programming of Stem Cells

**DOI:** 10.3390/ijms23041971

**Published:** 2022-02-10

**Authors:** Rüdiger Hardeland

**Affiliations:** Johann Friedrich Blumenbach Institute of Zoology and Anthropology, University of Göttingen, 37073 Göttingen, Germany; rhardel@gwdg.de; Tel.: +49-551-782270

**Keywords:** adipogenesis, cancer, chondrogenesis, inflammation, melatonin, neurogenesis, osteogenesis, stem cells

## Abstract

Melatonin interacts with various types of stem cells, in multiple ways that comprise stimulation of proliferation, maintenance of stemness and self-renewal, protection of survival, and programming toward functionally different cell lineages. These various properties are frequently intertwined but may not be always jointly present. Melatonin typically stimulates proliferation and transition to the mature cell type. For all sufficiently studied stem or progenitor cells, melatonin’s signaling pathways leading to expression of respective morphogenetic factors are discussed. The focus of this article will be laid on the aspect of programming, particularly in pluripotent cells. This is especially but not exclusively the case in neural stem cells (NSCs) and mesenchymal stem cells (MSCs). Concerning developmental bifurcations, decisions are not exclusively made by melatonin alone. In MSCs, melatonin promotes adipogenesis in a Wnt (Wingless-Integration-1)-independent mode, but chondrogenesis and osteogenesis Wnt-dependently. Melatonin upregulates Wnt, but not in the adipogenic lineage. This decision seems to depend on microenvironment and epigenetic memory. The decision for chondrogenesis instead of osteogenesis, both being Wnt-dependent, seems to involve fibroblast growth factor receptor 3. Stem cell-specific differences in melatonin and Wnt receptors, and contributions of transcription factors and noncoding RNAs are outlined, as well as possibilities and the medical importance of re-programming for transdifferentiation.

## 1. Introduction

Melatonin is a highly pleiotropic signaling molecule that acts on almost all organs and cell types [1]. Contrary to earlier belief, it is not only produced in the pineal gland and a very few other tissues but is synthesized in presumably all nondormant nucleate cells [2,3]. Whether melatonin is produced in those stem cells that are maintained in a resting stage within their respective niche has not yet been investigated. From a certain point of proliferative activation, this may be assumed to occur. The overall amounts of melatonin produced in the whole body exceed those in the pineal gland and in the circulation by orders of magnitude [1,4,5,6]. However, extrapineally formed melatonin is poorly released, at least in mammals, whereas the pineal gland plays a privileged role, as it is the major source of circulating melatonin and of the melatonin fraction that enters the third ventricle of the brain via the pineal recess [7,8]. On the other hand, melatonin also exerts paracrine and autocrine effects in tissues [9]. Therefore, processes taking place within a tissue seem to be influenced by both circulating and locally produced melatonin.

With regard to the importance of tissue renewal, for purposes of either rapid replacement of fast-growing cells, slow replacement in the course of aging, or of tissue repair after injury, the effects of melatonin on stem cells has gained particular attention. Moreover, numerous studies have demonstrated substantial influences by melatonin on lines of differentiation and fates of stem cells in various tissues, as will be outlined in detail. Most of these investigations have dealt with pluripotent cells from adult or adolescent animals or subjects, but a few studies have also reported effects on embryonal stem cells. Particular attention is also paid to actions on cancer stem cells, which reveal substantial differences to nontumor cells.

The present article will not discuss in detail all the different aspects of melatonin effects in connection with stem cells. For instance, a number of studies has dealt with adjunctive roles of melatonin for the success of transplantation or injection of stem cells at injured or dysfunctional sites [10,11,12,13,14,15,16,17,18]. Similarly, the antioxidant and anti-inflammatory properties of melatonin have been taken as a reason for protecting stem cells against oxidative and inflammatory insults [13,15,19,20,21,22,23,24,25,26,27]. Although this information is of high value, it largely exceeds the topic of stem cell programming. Such actions will only be briefly mentioned when appropriate in a broader context. Instead, focus will be laid on the signaling pathways that are steered by melatonin in the context of stem cell differentiation. These concern the initial involvement of the G protein-coupled membrane receptors, MT_1_ and MT_2_, and their primary transmission processes such as decreases of cAMP or activation of MAP kinase pathways [28], but also various downstream processes referred to as the extended signaling by melatonin, which may comprise activation of sirtuin-1 (SIRT1), or regulation of noncoding RNAs, such as miRNAs, lncRNAs, or circRNAs [29,30].

## 2. Pluripotent and Unipotent Stem Cells: Distinction between Programming and Self-Renewal

It is important to distinguish between the various types of stem cells, not only with regard to the organs in which they are residing, but also concerning the aspect of pluripotency. One extreme is that of totipotent cells present in the earliest embryonal stages. Their differentiation depends on developmental programs based on sequential gene expressions that are mainly influenced by neighboring cells. In this stage, a molecule like melatonin cannot be expected to interfere with differentiation processes, since this would disturb the course of development. However, this does not exclude some beneficial effects of melatonin, when present, regarding the protection against oxidative and other forms of cell stress. This assumption would be in line with findings on the protection of oocytes by melatonin [31,32,33,34,35], which was also described when present during in vitro maturation [36,37,38,39]. From a biological point of view, these results are not surprising, because the developing oocyte lives in the melatonin-containing environment of the follicle fluid, into which this compound is especially released by the oocyte and granulosa cells [36,40,41,42,43]. Although no programming by melatonin can be expected in oocytes and their early descendants, effects that exceed antioxidative protection are still possible. This is supported by the observation that oocyte maturation is enhanced by melatonin [40,41,42,43].

While oocytes are not stem cells in the proper sense, but rather stem cell precursors, the progenitors of spermatocytes are usually considered as spermatogonial stem cells (SSCs). In fact, actions by melatonin in SSCs, of a direct or indirect nature, have been described. These comprise protection against oxidative stress [44] and diabetes-induced dysfunction via protection of Leydig cells [45], promotion of differentiation to sperm-like cells [46], and enhanced proliferation via release of GDNF from Sertoli cells [47]. Regarding differentiation, SSCs are not pluripotent, but rather committed to develop towards sperms. However, the situation differs from others in which melatonin can influence the direction of differentiation among several alternate possibilities, as will be later discussed for MSCs (mesenchymal stem cells). Moreover, another mode of action seems to be excluded, namely, that of re-programming a stem cell. According to the requirements of sperm production, SSCs may be only stimulated to proliferate, to differentiate to sperm cells, and, with regard to the necessity for avoiding exhaustion of precursors, to warrant self-renewal of the SSC population by asymmetric cell division.

As several studies on SSCs have been conducted using whole tissue rather than isolated cells, a short remark shall be added. This concerns the question of whether proliferation in testes is sufficiently described on the basis of SSCs or whether contributions of pluripotent small embryonic-like stem cells exist, which may confound conclusions, as recently discussed [48,49,50].

The problem of presence or absence of pluripotency exists presumably for various other stem cells too. For example, satellite cells in skeletal muscle usually differentiate upon activation only to myoblasts and further to myocytes, which thereafter fuse to myotubes, the precursors of myofibers. The only alternative is asymmetric division to also generate satellite cells for self-renewal of the stem cell population. The lack of pluripotency may be seen as an advantage for muscular growth and regeneration. The decision for this developmental pathway has already taken place at an earlier step, when satellite cells are formed from pluripotent precursor stem cells [51,52]. Despite profound differences concerning dynamics and phase of differentiation during development, the situation is, in a sense, similar in cardiomyocyte precursor cells. The direct precursors, cardiomyoblasts, are already committed to this lineage, and only the preceding pluripotent cells can differentiate to other cell types. For example, pluripotent cardiac precursor cells have been shown to differentiate, under the influence of adipokines released from adipocytes, to mature adipocytes [53]. Cardiac stem cells and cardiomyocyte progenitors have been found to exist in epicardial stem cell niches [54], but their capacity to contribute to cell turnover or regeneration, which has traditionally been questioned, remains to be demonstrated.

Instead of elongating the list of unipotent stem cells, focus shall be laid on the profound difference to pluripotent stem cells concerning the possibilities of being influenced by melatonin. Unipotent and pluripotent stem cells may likewise be protected by melatonin against oxidative stress, ER stress, inflammation, mitochondrial dysfunction, and cell death, as is known for countless other cells. Moreover, both types may be stimulated to proliferate for purposes of self-renewal or expansion and be activated for undergoing the final differentiation step, which has, of course, to be demonstrated in each respective case. However, melatonin as a single factor does not directly exert, but only contributes to programming and also to re-programming in pluripotent stem cells, as will be outlined in the following sections, especially for MSCs (mesenchymal stem cells) and NSCs (neural stem cells). It is important to be aware that melatonin is not the only player in the differentiation processes, which are additionally under control of various transcription factors, humoral regulator molecules, exosome cargos, and intracellular noncoding RNAs, which can all be subject to other signaling mechanisms.

## 3. Melatonin and Unilateral Differentiation

Although predetermined cells that are committed to a single lineage are, under normal conditions, not subject to alternate programming, the processes of activation and differentiation are, in comparative terms, of interest to those which are taking place under conditions of melatonin-directed programming toward a specific route of development. Some of the mechanisms that lead to activation and entrance into the final differentiation step can be identical in unipotent and pluripotent stem cells, whereas others will determine, in the pluripotent cells, the direction of development.

Unilateral development is certainly the fate of spermatogonial stem cells (SSCs). Direct melatonin effects on SSCs have been rarely described. For instance, protection against palmitic acid-induced lipotoxicity has been reported [44]. Some of the effects are in line with frequent observations made with melatonin in its protective role, by preventing upregulation of the usual apoptosis-associated proteins including the transcription factor CHOP (C/EBP homologous protein), and of the ER stress markers p-IRE1 (=p-ERN1, phosphorylated ER to nucleus signaling 1 protein), p-PERK (phosphorylated protein kinase RNA-like endoplasmic reticulum kinase), and ATF4 (activating transcription factor 4). Importantly, palmitic acid-induced downregulation of Sox2 (sex determining region Y-box 2) and Oct4 (octamer-binding transcription factor 4) was prevented by melatonin, i.e., two factors of particular relevance to the functionality and differentiation of stem cells [44]. Another noteworthy finding of this study concerns the upregulation of SIRT1, with the consequences of p53 deacetylation and signaling via the downstream factor FoxO1 (forkhead box protein O1) [44]. Other effects of melatonin on SSCs are of an indirect nature and are observed in whole tissue or mixed cell cultures. Melatonin was shown to stimulate the expansion of goat SSCs by enhancing, in Sertoli cells, the secretion of GDNF (glial cell line-derived neurotrophic factor), which is, in this context, a decisive niche factor of Sertoli cells [47]. Another indirect effect of melatonin is mediated by Leydig cells. Goat SSCs were shown to differentiate to sperm or sperm-like cells under the influence of melatonin by enhancing testosterone release by Leydig cells [46]. A study in murine SSCs showed that melatonin protected Leydig cells against diabetes-induced deterioration, thereby preventing detrimental effects in the SSCs [45].

Formation of platelets from megakaryocytes represents another unilateral process of cell development. Melatonin was shown to promote thrombopoiesis by upregulating the ERK1/2 (extracellular signal regulated kinase 1/2) and Akt pathways [55]. In principle, both routes can be activated by different mechanisms that are initiated by the melatonin receptors, MT_1_ and MT_2_ [28]. In the case of megakaryocytes, the activation of PI3K (phosphoinositide 3-kinase) seems to be crucial for both ERK1/2 and Akt activations. On the one hand, Akt is a direct downstream factor of PI3K, whereas on the other hand, PI3K also upregulates DAPP1 (dual adaptor for phosphotyrosine and 3-phosphoinositides), which activates ERK1/2 [55].

Pancreatic stem cells (PSCs) have also been shown to be influenced by melatonin [56]. Again, effects via MT_2_ receptor and ERK activation were observed. However, melatonin was not found to initiate differentiation, but rather to stimulate PSC proliferation, maintaining the stem cell status, as indicated by upregulating the stem cell marker, nestin, via activation of the SMAD pathway (homolog of *Caenorhabditis* SMA = “small” worm phenotype and *Drosophila* MAD family = mothers against decapentaplegic) [56]. The self-renewal of PSCs is further enhanced by a positive feedback loop, in which nestin leads to the release of TGF-β1 (transforming growth factor-β1), which upregulates SMAD4 [56]. In a broader context, it should also be noted that TGF-β possesses additional anti-inflammatory properties [57,58,59], which may contribute to a protective maintenance of the PSC population.

Concerning the route of myocyte/myotube formation in skeletal muscle, different findings have been described for the consecutive steps of differentiation. Melatonin was reported to drive myogenic differentiation from mesenchymal stem cells [60]. Satellite cells present in the muscular niches have been found to be protected and stimulated to propagate in both chick embryos [61] and injured rat skeletal muscle [62]. However, conclusions on the regenerative potential of melatonin would have to solve a problem concerning effects on myoblasts, which represent the stage preceding that of the myocyte. In fact, C2C12 myoblasts were shown to be stimulated by melatonin to proliferate, but also to be inhibited in differentiating to myocytes [63]. This observation is in accordance with the downregulation of the transcription factor MyoD (myoblast determination protein), being otherwise a master regulator of skeletal muscle differentiation [64]. In addition to these findings, protection of myoblasts by melatonin has been repeatedly reported (e.g., refs. [65,66]), but these results are not directly related to differentiation.

The influence of melatonin on the formation of cardiomyocytes is even less understood. An early step of differentiation to heart cells seems to be positively influenced by melatonin, namely, the commitment of pluripotent embryonal stem cells to enter the cardiomyocytic cell lineage. Murine embryonal stem cells were shown to develop to cells expressing cardiac cell-specific genes, such as myosin heavy chains 6 and 7 [67]. Along with these changes, melatonin was shown to destabilize HIF-1α (hypoxia-induced factor 1α), but instead to upregulate HIF-2α [67]. The roles of these HIFs in cardiomyogenic differentiation are not fully understood. Although melatonin decreased HIF-1α expression, deletion of this factor was reported to prevent cardiomyogenesis and also HIF-2α stabilization [67]. Another aspect of interest concerns the involvement of SIRT1, which is upregulated under the influence of melatonin, reportedly depending on HIF-1α [67]. Effects of melatonin in cardiomyoblast maturation have not been sufficiently studied. To date, melatonin-controlled signaling mechanisms, including the role of SIRT1, have only been studied in the context of protection [68,69,70].

The role of melatonin in differentiation of epithelial and endothelial stem cells or progenitor cells has been poorly investigated. Beneficial effects on skin thickness in postmenopausal rats have been partially interpreted in terms of upregulation of putative stem cell markers, such as (i) c-Myc (cellular homolog of myelocytomatosis), which is involved in pluripotency, but has additional functions; (ii) FGF-β (fibroblast growth factor-β); (iii) the Wnt downstream factor β-catenin, which is involved in selective differentiation; and (iv) the receptor of SCF (stem cell factor), c-Kit (homolog of viral oncogene v-Kit), a receptor tyrosine kinase that is involved in numerous developmental processes [71]. Thus, conclusions are rather indirect and do not allow specific interpretations concerning cell types. With regard to endothelial stem cells, data on differentiation by melatonin are, again, limited, partially because of overlapping processes involving different cell types that can be engaged in vascularization and by pluripotency of earlier progenitor cells. In this place, only one study shall be briefly mentioned that has used “early outgrow” endothelial stem cells (eESCs). These were shown to be suitable for protective purposes upon renal ischemia. Pretreatment of eESCs with melatonin did not only warrant the renoprotective activity, which was absent in nontreated cells, but also stimulated migratory behavior of these cells and secretion of vascular EGF (epidermal growth factor) [11]. It may be assumed that the protective effects would have involved differentiation and maturation of the eESCs. 

## 4. Hair Follicle Stem Cells: A Lesson on Dose, Conditionality, and Stemness Maintenance

The skin contains various stem and progenitor cells. With regard to effects of melatonin, research has focused on two types, melanocyte progenitor cells and hair follicle stem cells (HFSCs), which are located in the bulge area of the hair follicle. In the former cell type, effects by melatonin that modulate pigmentation have been described [72], but these have not been related to differentiation-related processes. More details have been recently published on the latter cell type, showing profound actions of melatonin [73]. Although HFSCs are pluripotent, the alterations induced by melatonin do not lead to a change in the differentiation status [73], but, importantly, they involve signaling pathways that are relevant to programming and differentiation in various types of stem cells. The pluripotency of HFSCs is beyond any doubt, because they can differentiate in an ectopic environment to nerve cells, glial cells, smooth muscle cells, cardiomyocytes, or keratinocytes [74]. In the hair follicle, HFSCs mainly differentiate into cells of the dermal papilla, which produces the hair shaft, and cells of the dermal sheath [75]. In the skin, HFSCs can contribute to wound healing [76], perhaps via differentiation to dermal sheath cells [75]. Wound repair requires signaling via SDF-1α (stromal cell-derived factor, also known as the chemokine CXCL12), and its receptor CXCR4 [76].

Effects of melatonin on the hair follicle have been repeatedly described, e.g., in cashmere goats [77] and rabbits [78], but the interest was mostly focused on hair growth and not so much on differentiation. In the Cashmere goat, a seasonal breeder that produces a valuable fur, the influence of melatonin on hair growth is likely, from a fundamental point of view, as it is related to the formation of winter fur. Whether such results would be translatable to the poorly seasonal human remains uncertain. 

Interestingly, a factor of utmost importance for controlling the direction of differentiation processes is relevant to the actions of melatonin on HFSCs. This regulator is Wnt (a name derived from the *Drosophila* gene *wingless* and the murine virus integration gene *Int-1*). As will be discussed later, in the context of mesenchymal stem cells, presence or absence of Wnt signaling reflects a switch between differentiation routes. Wnt inhibits, via its receptor Fzd (Frizzled), a protein complex of downstream factors (axin/GSK3β, glycogen synthase kinase-3β), which degrades β-catenin (=catenin β). As a result of this inhibition, this key mediator of Wnt signaling accumulates. The Wnt pathway has been shown to be required for the initiation of hair growth [78,79,80].

The effects of melatonin on goat HFSCs revealed several surprises [73]. First, melatonin caused dose-dependently increases in levels of β1-catenin, up to a concentration of 0.5 mM, especially upon extended incubation. However, at 1 mM or higher, β1-catenin levels decreased. At optimal conditions for β1-catenin accumulation, upregulations of TF4 (transcription factor 4) and its interaction partner LEF1 (lymphoid enhancer-binding factor 1) were observed, which promoted the expressions of c-Myc, c-Jun, and cyclin D1, i.e., stimulators of cell cycle progression. Additionally, melatonin strongly upregulated BMP4 (bone morphogenetic protein 4), but also the internal BMP antagonist Noggin. Upregulation of Noggin is, however, relevant to stemness and serves the maintenance of pluripotency. This was confirmed by the expression of the homeobox protein Nanog, of Oct4 (organic cation/carnitine transporter 4), and the hematopoietic progenitor cell antigen CD34. In summary, the Wnt/β1-catenin pathway leads to a dual effect in the HFSCs, namely, the combination of cell proliferation and the maintenance of the pluripotent stem cell status, which is typically achieved by a step of asymmetric cell division. This discriminates the situation in HFSCs from the processes in several other stem cells, in which the Wnt/β-catenin route may also stimulate proliferation, but is associated with differentiation and, thus, the loss of stemness. However, it seems important to be aware that the seemingly identical players are, in fact, often different and cell type-specific. At least 19 isoforms of Wnt exist, and variants are also known for their receptors, co-receptors and downstream factors, such as the LEFs (lymphoid enhancer factors) and their interacting transcription factors [81].

## 5. Hematopoietic Stem and Progenitor Cells

In the previous section, cells have been already discussed that are forced to produce high quantities of differentiated cells. This is even more the case in hematopoietic stem and progenitor cells (HSPCs), however with the important difference that they give rise to numerous cell types, which requires repeated splitting of lineages based on respective molecular signals [82]. In brief, the most actual concepts indicate a primary bifurcation into an erythrocyte/megakaryocyte branch, which later splits into these two cell types, and a second branch that yields the rest of the blood cell types. This second branch splits into a lymphoid subbranch and another one that separates into dendritic cells and a monocyte/eosinophil/neutrophil lineage [82]. Further differentiation processes occur in the lymphoid subbranch and in the monocyte/eosinophil/neutrophil lineage, giving rise to the various known cell types and their functional variants. The factors governing the multiple differentiation processes are partially known, but a detailed outline of this highly complex matter would exceed the scope of this article, especially as these details have not been addressed in the melatonin-related literature. At least it should be mentioned that the development within the HSPC tree comprises both proliferation and differentiation that is associated with reductions of pluripotentiality. Moreover, the hematopoietic system displays a certain degree of flexibility that allows adaptation to changing demands of the organism.

In summary, the fate and regulation of HSPC descendants cannot be described in a few words and can neither be easily investigated as a whole, e.g., under the influence of a single factor like melatonin. Moreover, most respective information is largely restricted to effects of the circadian system, light and darkness, and a role of melatonin is only assumed with regard to its association with the scotophase [83,84,85,86]. While the circadian aspects concerning both oscillators and phase-specific increases of regulating factors are convincing, melatonin-related findings are only partly of interest. Light onset was found to stimulate the release of norepinephrine (NE), which downregulates CXCL12, and of TNFα, which causes increases in ROS. TNFα levels were associated with the rise of melatonin. While TNFα was concluded to stimulate HSPC differentiation, details concerning the bifurcations of HSPC development were not communicated. Melatonin was reported to upregulate the SCF receptor c-Kit, an important finding, as it indicates the support of stemness maintenance in bone marrow HSPCs during phases of proliferation [83]. This may be functionally paralleled by the increase of CXCL12, which favors HSPC homing to the bone marrow [86]. In the future, many more details on the branched differentiation routes and their determining factors would be required for a profound understanding.

## 6. Neural Stem Cells and Neurogenesis

### 6.1. Developmental Potential of Neural Stem and Progenitor Cells

Neural stem cells (NSCs) are the source of neurogenesis but can alternately differentiate to astrocytes and oligodendrocytes [87,88,89]. Importantly, neurogenesis is possible in some regions of the adult brain, mainly in the subgranular zone (SGZ) of the hippocampal dentate gyrus (DG) and in the subventricular zone (SVZ). In the course of neurogenesis, NSC populations and their derivatives called radial glial cells first expand by symmetric cell division, followed by asymmetric divisions that result in (a) an NSC that maintains stemness and the capacity of self-renewal, and (b) a neural progenitor cell that further develops into a neuron [87,90,91]. Radial glial cells may also develop into astrocytes, especially when neurogenesis stops [87,90]. According to actual knowledge, it is important to be aware of their heterogeneity that comprises states of quiescence and activation, intermediary subtypes, regional positioning within the niche, influences of the microenvironment, and variations according to differences in transcriptional and metabolic states [90,91,92]. This heterogeneity has not always been sufficiently considered, a fact that leads to the necessity of gradually omitting this aspect in the discussion of melatonin’s effects. 

Concerning the differentiation to oligodendrocytes, a peculiarity concerning their direct precursors, the NG2 glial cells, a separate discussion in Section 7 is required, as these cells do not only develop to oligodendrocytes, but also function as important regulators of microglia [59,93,94]. 

### 6.2. Reprogramming of Skin Fibroblasts and Other Cells to NSCs

The remarkable flexibility of NSCs, which is also reflected by their heterogeneity, allows other cell types to become reprogrammed for entering the neurogenic route of development. Even murine, porcine, and human fibroblasts were shown to be reprogrammed to NSCs [95,96,97], e.g., by combined overexpression of neural-specific transcription factors, such as BRN2 (brain-specific homeobox/POU domain protein 2), ASCL1 (achaete–scute family BHLH transcription factor-1), MYT1L (myelin transcription factor 1 like), and another basis helix–loop–helix protein, NeuroD1 [95,96]. The combination of ASCL1 with microRNAs (miR-9/9* and miR-124) was shown to be likewise effective in reprogramming [96]. In human skin fibroblasts, this was also achieved by combinations of various inhibitors and activators of known signaling pathways, but strongly supported by a substantial contribution of melatonin [97]. This finding was entirely convincing, since melatonin upregulates the required factors BRN2, ASCL1, and MYT1L as well as several other neuronal proteins such as DCX (doublecortin), Sox2 (sex determining region Y-box 2), and NeuN (neuronal nuclei) [97]. However, it should be noted that not all of these proteins are exclusively neurally expressed but are also present in other cells. For instance, Sox2 is involved in the maintenance of pluripotency and differentiation processes of various stem cell types and is particularly relevant to cancer stem cells [98]. MYT1L was shown to be also expressed in the oligodendrocyte lineage [99]. 

Reprogramming to NSCs and neurons deriving thereof has also been demonstrated for MSCs [100,101,102]. In particular, amniotic fluid MSCs [100] and dental pulp MSCs [102] were shown to differentiate into dopaminergic neurons under the influence of mixtures of neurotrophic factors and/or melatonin, findings of particular interest to the therapy of Parkinson’s disease. In the MSCs from amniotic fluid, melatonin induced changes in surface markers, reducing the expressions of CD29, CD45, CD73, CD90, and CD105 [100]. In the dental pulp MSCs (DPMSCs), melatonin caused upregulations in neuronal markers and in tyrosine hydroxylase [102]. Additionally, it promoted the phosphorylation of a decisive transcription factor in Hippo signaling, YAP (Yes-associated protein), at Y357, a change that causes cytoplasmic retention and proteasomal degradation of this factor. Since unphosphorylated YAP, in conjunction with its association partner TAZ (transcriptional co-activator with PDZ binding motif), supports stemness and self-renewal [103], the also observed reduction of the stem cell marker nestin [102] is highly plausible. The effect of melatonin on Hippo/YAP signaling is also of interest to other properties and actions of melatonin, because of a considerable overlap and antagonism. Apart from the fact that the Hippo pathway is involved in the regulation of the innate immune system [103], it also functions as part of the stress response in stem cells [104]. Hypoxia and oxidative stress have been shown to upregulate this pathway, which leads to the induction of proapoptotic genes and autophagy [104]. Therefore, melatonin may counteract Hippo signaling at multiple levels because of its anti-inflammatory, antioxidant, and anti-apoptotic properties. Finally, the YAP/TAZ-mediated function of maintaining self-renewal is a substantial advantage to cancer stem cells [105]. In the future, this may be more specifically considered in the context of melatonin’s oncostatic actions, to which YAP phosphorylation may contribute.

### 6.3. Neurogenesis from NSCs/NSPCs 

Melatonin-induced stimulation of NSCs or NSPCs (neural stem/progenitor cells) to proliferate and/or to differentiate into neurons has been amply documented [19,20,88,106,107,108,109,110,111,112,113,114,115,116,117,118,119,120,121,122,123,124,125,126,127,128,129,130,131,132,133,134,135,136,137,138,139,140,141] (Table 1). However, melatonin was also occasionally reported to reduce NSC proliferation, e.g., in a normal light/dark cycle, in which cell division was found to be reduced in the scotophase [142]. Moreover, the survival of NSCs/NSPCs has been repeatedly reported to be promoted by melatonin, under various conditions [10,13,15,19,20,110,113,121,122,126,139,140]. The state of the art until 2015 was summarized in an excellent review by Chu et al., which has addressed some aspects of signaling [88]. Many other details are found in a recent review of broader scope [27]. While melatonin supported the differentiation to neurons, it either remained without effect under the same conditions in astrocytes [19] or even suppressed the astrocytic lineage [110], according to the criterion of GFAP (glial fibrillary acidic protein) expression. 

Concerning survival, proliferation, and signaling, it is, however, important to perceive the differences between unchallenged and challenged conditions. In the absence of toxicological, endocrinological, surgical, or inflammatory challenges, the effects of melatonin may more correctly reflect its natural potential of regulation. Protective actions observed under challenged condition are certainly of great value for medical applications. However, they are also affected by the problem that the observed protection may only reflect the attenuation of the causes of damage, but not the normal developmental processes of NSPC programming. 

As will be also reported in the section on MSCs, the natural programming processes in NSCs and NPCs are usually mediated via melatonin’s membrane receptors, MT_1_ and/or MT_2_ [19,27,88,107,109,139]. The most frequently observed signal transmission is that of the canonical ERK/MAPK pathway [19,107,114,115,116]. Another repeatedly reported downstream pathway is that of PI3K/AKT signaling [20,27,88,112,117]. These findings are in line with those on programming of other stem cells, especially MSCs, as will be discussed in Section 8.

The influence of melatonin on survival, proliferation, and differentiation of NSPCs is of utmost medical importance, as this offers new concepts of treatment in neurodegenerative diseases and in various forms of brain and spine injuries. 

The transfer of NSPCs to dysfunctional or damaged sites has often faced problems of poor survival and maldifferentiation. At least at the preclinical level, the use of melatonin has repeatedly overcome these difficulties and brought about considerable improvements [27,139,140,141,143]. These observations are in accordance with the multiple results on NSC protection, proliferation, and differentiation both in vivo and in vitro (Table 1).

Additionally, remarkable findings shall be underlined showing that site-specific development and neuronal differentiation can be also achieved by the transfer of stem cells other than NSCs to areas of the CNS. This has been demonstrated with bone marrow MSCs [144], adipose tissue-derived MSCs [145], amniotic fluid MSCs [100], and dental pulp MSCs [102]. In the two latter cases, the MSCs were more specifically differentiated to dopaminergic cells, as judged by the expression of tyrosine hydroxylase [100,102]. From a fundamental point of view, this sheds light on the role of the tissue-specific microenvironment, which may include the locally residing neurons and astrocytes, their secreted factors, and presumably also the composition of the extracellular matrix. The secretion of neurotrophic factors such as BDNF and GDNF taking place under the influence of melatonin (cf. Table 1) may support the desired fate of the transferred stem cells. The role of the extracellular matrix in melatonin-supported stem cell differentiation has been mostly studied in MSCs, but it may also be relevant to NSCs. Several studies beyond melatonin research may be indicative for this assumption. The observation that transplantation of stem cells (either embryonal NSCs or MSCs) close to a site of brain injury (surgical or ischemic) leads to modifications of the extracellular matrix that results in a biobridge, which facilitates the migration of NSCs from neurogenic niches to the site of damage, whereby the endogenous NSCs can replace the transplanted cells [146,147,148,149,150]. Such processes as well as the modification of the extracellular matrix should also be considered in future studies on stem cell-mediated repair under the influence of melatonin.

## 7. NG2 Glia and Oligodendrocytes, a Field of Future Perspectives

Although NSCs can also differentiate to oligodendrocytes, this has been rarely investigated under the influence of melatonin. Enhanced differentiation of murine cortical NSCs to oligodendrocytes has been reported to occur upon melatonin treatment [106]. This may have been overlooked or not been followed in other studies, which have not been interested in white matter. Another investigation that focused on damage of white matter after focal cerebral ischemia described beneficial effects of melatonin, which comprised upregulation of NG2 (Neural–Glial 2) [151], a marker of NG2 glia, which had previously only been regarded as so-called polydendrocytes, i.e., oligodendrocyte precursors. However, the actual view is that of a fourth category of glia, termed NG2 glia, which has specific functions in the regulation of microglial activities [93,94,152,153]. With regard to the profound roles of microglia in both inflammatory insults and protection within the CNS as well as the influence of melatonin in maintaining the balance of microglial polarization and activities [59], the role of NG2 should come into the focus of melatonin research. The actions of melatonin in this cell lineage can be assumed to be of importance for both inflammatory processes in the brain, including the prevention of inflammatory damage to NSCs, and to the maintenance of functional oligodendrocytes, e.g., in the context of multiple sclerosis.

## 8. Mesenchymal Stem Cells

### 8.1. The Numerous Variants of MSCs

Mesenchymal stem cells (MSCs) are by no means a homogeneous entity. Although their subtypes share numerous properties and overlap regarding their differentiation potential, they also differ with regard to their properties when investigated either in their natural microenvironment or when studied under the influence of melatonin. Their differences, which can, however, partially be overcome by appropriate experimentation, primarily depend on their tissue-specific niches and microenvironmentally determined properties, which result in deviating expressions of important determination factors. A most striking difference concerns the expression of Wnt and its downstream factor β-catenin, as will be addressed in more detail in Section 8.2. The following MSC subtypes are usually discriminated according to their sources [154], which allows gradual distinctions of their properties, but also comprises similarities. The main subtypes, listed here according to origin, carry different surface markers (mentioned for human cells in braces): MSCs from bone marrow (SH2 (Src homology-2), SH3, CD29, CD44, CD49e, CD71, CD73, CD90, CD105, CD106, CD166, CD120a, CD124); synovial fluid (CD10, CD166, CD44, CD54, CD90, CD105, CD147, D7-FIB (D7-fibroblast antigen), STRO-1 antigen (STRO = mesenchyme)); adipose tissue (CD13, CD29, CD44, CD73, CD90, CD105, CD166, HLA-I, HLA-ABC); amniotic fluid (SH2, SH3, SH4, CD29, CD44, CD49, CD54, CD58, CD71, CD73, CD90, CD105, CD123, CD166, HLA-ABC); umbilical cord and cord blood (CK8 (cytokeratin-8), CK18, CK19, CD10, CD13, CD29, CD44, CD73, CD90, CD105, CD106, HLA-I, HLA-II); Wharton’s jelly (CD13, CD29, CD44, CD73, CD90, CD105, HLA-I); dental pulp (CD29, CD44, CD90, CD105, SH2, SH3, HLA-DR, CD117, CD146); skin (CD90, CD73, CD105, SSEA4 (stage-specific embryonic antigen-4)); salivary gland (CD13, CD29, CD44, CD49f, CD90, CD104, p75NGFR (p75 nerve growth factor receptor), β2-microglobulin, CD130); salivary gland (CD13, CD29, CD44, CD49f, CD90, CD104, p75NGFR, β2-microglobulin, CD130); and placenta (CD29, CD44, CD73, CD90, CD105). While some of these markers, such as CD44 and CD90, are generally MSC-specific or present in the majority of subtypes, such as CD29, CD73, and CD105, others and combinations of them are suitable for discrimination. Similarities also reveal some properties shared by subtypes. For instance, MSCs from bone marrow, adipose tissue, umbilical cord, salivary gland, and dental pulp can all enter chondrogenic, osteogenic, or adipogenic lineages [154]. In the cases of Wharton’s jelly and amniotic fluid, only osteogenic and adipogenic properties have been demonstrated, but the absence of chondrogenicity may only reflect a lack of specific investigation, as chondrogenicity and osteogenicity are mechanistically tightly coupled [60,155]. Additional properties such as a neurogenic potential observed in other subtypes should not be judged as being exclusive, since this may, again, only reflect gaps of experimental approaches. Nevertheless, the effects of melatonin in programming directed differentiation under appropriate conditions reveal differences in suitability of the respective subtypes. The interplay of melatonin and other differentiation/determination factors will be discussed in the subsequent subsections.

### 8.2. Presence or Absence of Wnt/β-Catenin Signaling: An Important Switch for Melatonergic Actions

Wnt signaling has been found to be a decisive process for the selective differentiation of stem cells. Its presence or absence determines the development into specific cell types and prevents development to others [156,157]. This is also of importance for the programming of MSCs by melatonin [60]. Wnt proteins are morphogens that may act as diffusible paracrine mediators [157] or in cell-to-cell transfer via specialized filopodia [157,158]. They participate in the self-organization of tissues and in regulation of numerous processes. In this role, they are influenced by the microenvironment, and, importantly, their signaling can be stimulated either by factors that promote Wnt formation and secretion, such as TGF-β [159], or by indirect activators. For example, R-spondins can bind to LGRs (LGR4, 5, or 6; leucine-rich repeat-containing G protein-coupled receptors) and eliminate endogenous Wnt receptor (Fzd) antagonists such as Strps, Wifs, and Znrfs [160,161,162]. However, the situation regarding such a multifunctional system involved in the fate of numerous different cell types is inevitably highly complex. Apart from the fact that all the regulators mentioned exist in several subforms, this is even more the case for the Wnt proteins, which exist in at least 19 variants [157,163], and also for their receptor, Fzd, of which 10 subforms are known [163]. 

With regard to such complexity and variability, which concerns all players, it seems highly unlikely that Wnt signaling as related to melatonin is precisely the same in the different MSC-descendant cell lineages, even though it may be based on the same principle. Differences in tissue or niche-specific microenvironments and subform expressions have to be taken into account when comparing the respective experimental results. 

Concerning the relationship between Wnt/β-catenin signaling and melatonin, a positive relationship has been repeatedly documented [60,73,155,164,165,166,167,168]. However, it is important to distinguish between synergistic actions and direct regulation of the Wnt pathway by melatonin. This is not always easy to decide on the basis of all results, which led to the conclusion of melatonin being upstream of Wnt. For instance, blocking of a melatonin effect by using an inhibitor of a late step in the pathway does not yet allow conclusions regarding Wnt stimulation by melatonin, because Wnt activity might alternatively only provide a precondition for melatonin’s action. As an example, suppression of a melatonin effect by XAV-939 [168] may not allow such a conclusion. This agent inhibits tankyrases 1 and 2, which leads to the upregulation of the axin–GSK3β complex that promotes the degradation of β-catenin. The same reservation has to be made for application of the β-catenin inhibitor ICG-001 [164]. For logical reasons, downstream inhibitions do not tell us anything about Wnt protein activity. Another logical problem arises when an intervention such as ovariectomy leads to the downregulation of a stimulatory factor like HGF (hepatic growth factor), which is, under melatonin treatment, restored [155]. In such a case, the melatonin effect may have originated in the restoration of HGF levels rather than in a direct action on Wnt. However, direct upregulation of Wnt by melatonin has also been observed. For instance, melatonin has been reported to induce Wnt4 expression through the ERK1/2-Pax2-Egr1 pathway [165]. As ERK1/2 activation is a canonical pathway of melatonergic signaling, this may, in fact, reflect a direct action of melatonin on Wnt. Nevertheless, as this study was conducted under inflammatory conditions, the findings might require some dissection from a relief from inflammatory/oxidative stress due to anti-inflammatory/antioxidant properties of melatonin [30,169]. Finally, with some caution, a canonical melatonin receptor-mediated effect on Wnt expression or activity may be assumed. However, with regard to the importance of this conclusion, more direct evidence from studies in the absence of challenges would be highly desired.

Whatever the precise relationship between melatonin and Wnt is, it can be stated with certainty that presence or absence of Wnt is decisive for some aspects of selective MSC differentiation, as will be outlined in detail in the following Section 8.3, Section 8.4 and Section 8.5. The absence of Wnt signaling is a precondition for adipogenic differentiation, whereas its presence is a requirement for chondrogenic and osteogenic [60] as well as—ectopically—neurogenic differentiation [163,170]. However, as Wnt activity is required for multiple routes of differentiation, additional factors or isoforms must be involved in the developmental bifurcations. 

### 8.3. Melatonin without Wnt Signaling: Adipogenesis

If melatonin is assumed to stimulate Wnt expression and signaling, the question remains why the absence of Wnt is a requirement of adipogenic differentiation under the influence of melatonin, as reported [60]. This is even more problematic, as melatonin was shown to be, in bone marrow MSCs, a negative regulator of adipogenesis, which suppresses the key adipogenic transcription factors C/EBPβ (CCAAT/enhancer-binding protein-β) and PPAR-γ2 (peroxisome proliferator-activated receptor-γ2), and also downregulates the adipocyte markers aP2 (adipocyte protein 2) and adiponectin [155,171,172]. This problem of differences between bone marrow (BMSCs) and adipose-tissue-derived MSCs (ADMSCs) has been tried to be explained by a tissue-specific epigenetic memory. The CpG islands in promoters relevant to either osteogenesis or adipogenesis were found to be differentially methylated; *Runx2* promoters in BMSCs were hypomethylated in BMSCs but hypermethylated in ADMSCs, whereas *PPARγ* promoters of BMSCs were hypermethylated but hypomethylated in ADMSCs [173]. This would mean that the osteogenic factor Runx2 (runt-related transcription factor 2) is suppressed in ADMSCs, whereas PPARγ is low in BMSCs, but not in ADMSCs. If this conclusion is correct, this could mean that the also assumed downregulation of PPARγ by melatonin in ADMSCs (cf. Ref. [60]) might be a misinterpretation of data from BMSCs that should not be translated to ADMSCs. This argument concerning high expression of PPARγ in ADMSCs would be in accordance with the general consideration of this factor as an adipocyte marker [155]. However, these epigenetic differences in DNA methylation of osteogenic and adipogenic factors do not yet explain the difference in Wnt signaling. The solution of this problem may be sought in findings concerning a relationship between DNA hypermethylation and reduced Wnt signaling in ADMSCs [174,175,176]. Although these results have not been made in a melatonin-related context, but rather in that of diabetes and advanced glycation end products, they may be taken as a proof of principle. Additionally, they are of interest to osteoporotic adipogenesis and the demand for reactivating osteogenesis (cf. Section 8.4).

### 8.4. Melatonin Effects in the Presence of Wnt: Chondrogenesis and Osteogenesis

Under the condition that Wnt signaling is active, the routes of chondrogenesis and osteogenesis are favored. This has been preferentially studied in BMSCs, but reprogramming of MSCs of other origin is also possible. In BMSCs, the subform Wnt4 has been shown to be involved in osteogenesis [165,177,178]. However, other subforms, namely Wnt3A, Wnt4, Wnt 7A, Wnt10A and Wnt11, were also reported to be expressed in BMSCs [179]. When upregulated by factors other than melatonin, osteogenesis can be stimulated via Wnt4 in MSCs not derived from bone marrow, e.g., in ADMSCs [180], myoblasts [181], inflamed dental pulp MSCs [182], and umbilical MSCs [183]. Thus, a particular association of this subform with osteogenesis seems likely. Nevertheless, osteogenic properties of other Wnt subforms should not be excluded and have, especially, been demonstrated for Wnt3a, which has even been directly applied to sites of desired bone formation [184,185,186].

The Wnt-dependent differentiation routes of chondrogenesis and osteogenesis are, in a sense, intertwined, as formation of cartilage usually precedes that of bone, but these processes have to be steered in a functionally favorable way, with a necessity of switching when desired between the two modes of development. As both chondrogenesis and osteogenesis are stimulated by melatonin [60], the switch would be difficult to be explained on the basis of this regulator. In fact, such a switch was ascribed to FGFR3 (fibroblast growth factor receptor 3) [187]. *Fgfr3* deficiency in chondrocytes was shown to cause osteogenesis, with upregulation of *Ihh* (Indian hedgehog), *Bmp2*, *Bmp4*, *Bmp7*, but also *Wnt4*, and *Tgf-β1*, and downregulation of the BMP antagonist, *Noggin* [187]. This finding appeared rather convincing, but the problem remained how FGF and FGFR3 are regulated. This starts with an unusual complexity, as at least, 23 subforms of FGF can be distinguished, and many of them have been poorly studied. According to actual knowledge, the many subforms act via four tyrosine kinase receptor subtypes, FGFR1–4. Moreover, FGFs are rarely found as freely diffusible molecules, but are rather bound to heparan sulfates and other proteoglycans or to FGF receptors. Thus, FGFs have been concluded to mainly act as autocrine factors and become active as primarily matrix-bound ligands [188]. More recently, a mechanism of FGF activity control has been discovered [189]. FGFRs were shown to shed their ectodomains, which retain their capacity of binding FGFs. This was assumed to allow depletion of cell surface-bound FGFs. Ectodomain shedding was demonstrated to require tyrosine kinase activity and PKC (protein kinase C) activation. The physiological activators of shedding have not yet been identified in the case of FGFs, but factors involved in ectodomain shedding of other receptors have been discussed, among them TNFα (tumor necrosis factor-α), TGF-α, L-selectin, and HB-EGF (heparin-binding epidermal growth factor-like growth factor) [189]. Moreover, it is still unclear to which of FGF and FGFR subforms the described mechanism is applicable. Thus, the identification of the complete physiological signaling pathway of the chondrogenic-to-osteogenic switch requires further investigation. Nevertheless, the impression remains that the switch is strongly dependent on the microenvironment including the extracellular matrix.

The chondrogenic actions of melatonin seem to differ from its osteogenic signaling, as the latter depends on the receptor MT_2_, whereas both MT_1_ and MT_2_ were reportedly expressed in chondrocytes [190]. Activation of chondrogenesis in the presence of Wnt and melatonin is associated with the upregulation of several cartilage markers and morphogenetic factors, such as GAG (glucosaminoglycan), Col2A1 (collagen type II, A1), Col10A1 (collagen type X, A1), ACAN (aggrecan), SRY (sex determining region Y), Sox9, Runx2, and BMP2 [60,155,190]. Notably, BMP2 is, despite its name as a bone morphogenetic protein, not a factor specific for osteogenesis. Moreover, it should be emphasized that Sox9 can prevent the transdifferentiation of chondrocytes to osteoblasts [191], whereas Runx2 favors both chondro- and osteogenic differentiation [192]. However, such an action of Sox9 occurs after the switch towards chondrogenesis. In addition to Sox9, other subforms such as Sox5 and Sox6 participate, but their relationship to melatonin has remained unclear. The precise signaling routes of melatonin-promoted chondrogenesis require further clarification. The involvement of MT_1_ and MT_2_ may indicate that the primary signaling could occur via the MEK1/2-ERK1/2 pathway. The subsequent steps toward Wnt activation have remained unclear, especially as several subforms of Wnt and Wnt receptors are involved, partially with opposite actions during the progression of chondrogenesis. With regard to Wnt receptors, both canonical and noncanonical routes seem to play a role. Moreover, the upregulation of BMP9 antagonists such as Noggin and Chordin, which prevent transition to osteogenesis, await further investigation.

A specific aspect of chondrocyte biology concerns the formation of hypertrophic chondrocytes. These represent an end stage of this cell lineage and allow the transition to bone formation by transforming into osteoprogenitors, especially in endochondral bone development [191,192,193]. However, in a pathophysiological context, excessive chondrocyte hypertrophy is also involved in osteoarthritis [192,194]. Hypertrophic chondrocytes gradually upregulate Ihh and Col10A1, but downregulate Sox9, which leads to the expression of osteoblast-specific genes, such as *Mmp14* (matrix metalloproteinase 14), *Ibsp* (integrin binding sialoprotein, *alias* bone sialoprotein II), and *Vegfa* (vascular endothelial growth factor A) [191,192]. A certain fraction of hypertrophic chondrocytes undergo apoptosis, leaving a scaffold in the cartilage matrix for entrance of other cells that remodel and calcify the cartilage [191]. Importantly, the transformation to hypertrophic chondrocytes is also promoted by melatonin via Wnt4/β-catenin signaling [168], which is in line with its general pro-osteogenic properties. 

**Figure 1:**
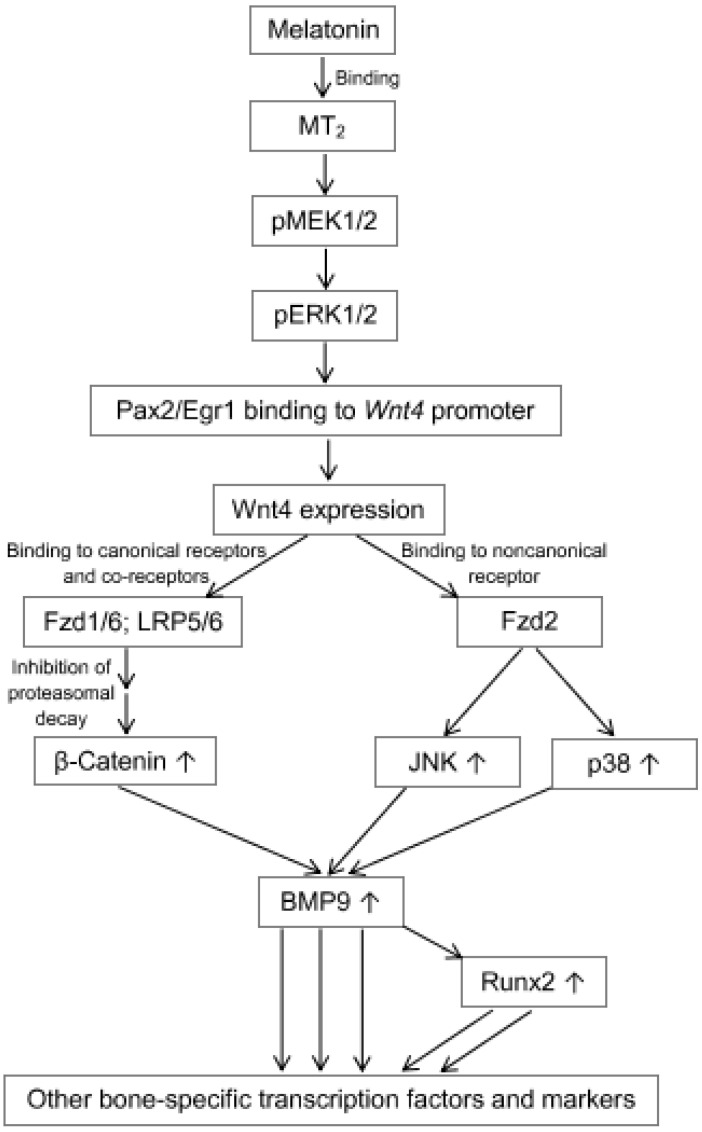
Osteogenic signaling of melatonin via Wnt4 expression and both canonical and noncanonical Wnt pathways. Some of the depicted relationships are based on ref. [165]. Abbreviations: BMP9, bone morphogenetic protein 9; Egr1, early growth response protein 1; Fzd, Frizzled; JNK, c-Jun-N-terminal kinase; LRP, low density lipoprotein receptor-related protein; pERK1/2, phosphorylated extracellular signal-regulated kinase-1/2; pMEK1/2, phosphorylated MAPK/ERK kinase-1/2; Pax2, paired box 2; Runx2, runt-related transcription factor 2. Symbol: ↑ upregulation.

The osteogenic differentiation of MSCs, frequently studied in BMSCs, is most amply documented within the area of morphogenetic signaling by melatonin [22,60,122,164,165,166,167,172,177,195,196,197,198,199,200,201,202,203,204,205,206]. In one case, an osteogenic potential of BMSCs was reported to be rescued by melatonin from titanium-induced impairment via HIF-1α stabilization and SIRT1 signaling [204], but this may be explained by counteraction against the metal-promoted oxidative stress rather than by a primary promotion of osteogenesis. Bone-specific effects of melatonin in the narrow sense are generally mediated by the receptor MT_2_ [165,167,195,196,199], which has been shown to act in BMSCs via the canonical MEK1/2-ERK1/2 pathway [165,199]. The alternate possibility of ERK1/2 phosphorylation via Ras/Ref has, at least, not been convincingly demonstrated in these cells. According to actual interpretations, the subsequent signaling involves the transcription factors Pax2 (paired box 2) and its interaction partner, Egr1 (early growth response protein 1), which initiate the expression of Wnt4 (Figure 1). In the respective study [165], the Egr subtype had not been identified, but the expression of Egr1 in BMSCs has been recently documented elsewhere [207]. Wnt4 signaling occurs via both canonical and noncanonical pathways. In the first case, Wnt4 acts via the Fzd1 and Fzd6 receptors with their co-receptors LRP5 and LRP6 (low density lipoprotein receptor-related proteins 5 and 6), which cause, via the mechanism described in Section 4, increased levels of β-catenin. In the second route, Wnt4 acts via Fzd2 that activates the JNK (c-Jun-N-terminal kinase) and p38 pathways. These pathways jointly stimulate the upregulation of two decisive bone morphogenetic factors, BMP9 and its most important downstream mediator [208], Runx2. Additionally, the differentiation-related transcription factor Osx (osterix) is upregulated, a process that would require further clarification. As a result of these differentiation-promoting signals, several bone/osteoblast-specific proteins are expressed, such as ALP (alkaline phosphatase) [195,197,198,206,209], OSP (=OPN, osteopontin) [196,206], OCN (osteocalcin) [196,198], and OPG (osteoprogeterin) [210]. With regard to BMP9 signaling in BMSCs and bone, actions via Runx2 and its more universal mediator, Hey1 (hairy/enhancer-of-split with YRPW motif protein 1), are of particular relevance. Various genes regulated by BMP9 have been recently identified and summarized [211].

Several details related to BMSC signaling that have not been so much in focus shall be also briefly mentioned here. As increasingly perceived, gene regulation comprises actions by noncoding RNAs, and this includes actions of melatonin [30]. While some lncRNAs and miRNAs were shown to suppress osteogenesis [167,178], others support it. With regard to melatonin-stimulated osteogenesis, the lncRNA H19 was reported to facilitate this [167]. The mode of action was found to consist in the sponging of the suppressive miR-541-3p, which targets the *Apn* (adiponectin) mRNA. Thus, the removal of miR-541-3p leads to increased expression of APN, a factor that signals to MSCs fully loaded fat stores, in other words, the unnecessity of adipocyte generation. Thus, APN may contribute to the switching from adipogenesis to osteogenesis (cf. Section 8.3). Upregulation of APN has been interpreted as a signal for Wnt expression and β-catenin accumulation [167]. Circular RNAs (circRNAs) represent another category of miRNA sponges. In the context of ovariectomy-induced decreases of melatonin and induction of osteoporosis, the circRNA circ_0003865 was found to sponge the pro-osteogenic miR-3653-3p, which targets *Gas1* (growth arrest specific protein 1) mRNA, a factor that blocks osteogenesis. Melatonin was shown to upregulate in BMSCs miR-3653-3p, thereby inhibiting GAS1 expression [206].

Finally, a deviation of melatonin-supported osteogenesis shall be mentioned that concerns BMSCs in the context of growing antlers in the Sika deer [212]. This investigation is of interest, as it has to be seen in the context of melatonin’s role in seasonality. Contrary to all other findings in humans, laboratory rodents, and chicken, melatonergic signaling in antler BMSCs and bone was reported to not be mediated by MT_2_, but rather by MT_1_. Moreover, MT_1_ was related in this study to the upregulation of IGF1 (insulin-like growth factor) and its receptor IGF-1R. The peculiarities of very rapid growth of antlers may be assumed to be responsible for these deviations.

### 8.5. Neurogenesis from MSCs with Noncanonical Wnt Signaling

It is of utmost importance to consider the pluripotency of MSCs with regard to the possibility of reprogramming to other stem cell types, which allows alternate ectopic differentiation and opens remarkable new ways of tissue repair. For instance, DPMSCs, ADMSCs, and BMSCs have been shown to be capable of becoming transdifferentiated to neuronal precursors and yield site-typical neurons [102,145,213]. Some respective findings have already been mentioned in Section 6.2, including the role of Hippo signaling. Here, the involvement of Wnt signaling is emphasized. Neurogenic differentiation from both ASMSCs and BMSCs has been shown to also require Wnt expression and activation of the JNK pathway [213]. In both cases, upregulation of Wnt 5a was found to be associated with neurogenesis. In the case of BMSCs, Wnt4 and Wnt1 were also shown to be upregulated during neurogenic induction. Another important difference to chondrogenic and osteogenic differentiation concerns the upregulation of the Wnt receptor Fzd3 [213]. This latter finding indicates that Wnt5a may act in this case via a canonical pathway. However, the also observed upregulation of the JNK pathway speaks for noncanonical signaling, since this latter pathway is typically activated by Wnt binding to ROR2 [157] (receptor tyrosine kinase-like orphan receptor 2; not to be confused with other orphan receptors, RORα and β, once discussed as nuclear melatonin receptors). A further complication results from the observation that Wnt5a signaling can inhibit the β-catenin-mediated pathways activated by other Wnt subforms [214,215]. The role of melatonin in Wnt5a regulation of neurogenic differentiation is not yet settled. However, this information may be important to further studies on this topic of MSC ectopic transdifferentiation in the CNS. 

### 8.6. Reprogramming and Therapeutic Use of MSCs

Reprogramming of stem cells is an issue of the highest interest for purposes of tissue repair and replacement, which will gain considerable value in future therapies. This has already been and will be more often applied by the transfer of stem cells to sites of damage. The role of melatonin can be sought in both the protection of the transferred stem cells and the support of differentiation to the desired cell type. The protective aspect has been comprehensively reviewed [13,27]. Some promising studies of this type shall be mentioned here.

Improved wound healing in the skin was achieved by transplanting umbilical cord MSCs with support by melatonin treatment [18]. Hepatic tissue repair for the purpose of ameliorating liver fibrosis was performed with melatonin-preconditioned BMSCs [14] or dental pulp MSCs [12]. Most of the actions by melatonin were related to its anti-inflammatory actions, but in the DPMSCs, hepatic differentiation was associated with an upregulation of BMP2 [12], i.e., a bone morphogenetic protein subtype that is not bone-specific. Hepatic MSCs, which also exist, were not applied in this study. However, fetal MSCs from liver were used to restore ovarian function in premature ovarian failure or insufficiency, using an in vivo model of follicular development and a human in vitro model of granulosa cells [216]. Protective effects observed were associated with upregulation of the MT_1_ receptor, which should have a functional relationship to natural ovarian melatonin synthesis [217,218], as well as increased JNK1 and AMPK signaling as well as PCNA (proliferating cell nuclear antigen) expression, an indicator of cell proliferation [216]. Several studies have dealt with tissue repair after kidney ischemia/reperfusion using injections of MSCs or endothelial progenitor cells pretreated with melatonin [10,11,219]. Engraftment of melatonin-preconditioned BMSCs was successfully used for renal regeneration in a rat model of chronic kidney disease [17]. Again, the antioxidant and anti-inflammatory actions of melatonin contributed strongly to the observed effects.

With regard to the high importance of bone tissue repair, after fracture as well as in osteoporosis, transplantation of cells that differentiate to osteoblasts is of particular interest. C3H10T1/2 pluripotent MSCs were shown to express osteogenic markers and BMP9, not only in cell cultures, but also in embryonic limbs and in ectopic, subcutaneous transplants, whereby the combination of melatonin and BMP9 proved to be particularly efficient [201]. Another field of special relevance is that of neuroprotection. The above-mentioned possibility of transdifferentiating MSCs to neuronal precursor cells (Section 8.5) has also prompted researchers to transplant suitable cells to the CNS and to promote the outcome by melatonin. BMSCs pretreated with melatonin were transplanted to treat damage by focal cerebral ischemia, with a favorable outcome, especially with regard to melatonin-dependent survival and functionality of the transplanted cells [144]. In another study using an Alzheimer’s disease model, rats were intravenously injected with ADMSCs that had been pretreated with melatonin [145]. In fact, ADMSCs, whether melatonin-treated or not, migrated into the CNS. However, only those cells that had been exposed to melatonin exhibited favorable effects and reported decreases in amyloid-β and cognitive improvements.

Although these investigations have still remained rather fragmentary, they are also highly encouraging. After further technical refinements, melatonin-aided transplantation of stem cells will presumably have a promising future.

### 8.7. MSC-Derived Exosomes with Protective, Anti-Inflammatory, and Antioxidant Effects

Melatonin has been shown to modulate the cargo composition of exosomes and, thereby, the regulatory potential of these vesicles [3,29,220,221]. This was impressively confirmed for MSCs, as recently summarized with a focus on tumor biology [222]. Importantly, MSCs were found to release relatively high amounts of exosomes [223]. MSC-derived exosomes are of particular interest, because they can have immune suppressive properties [224]. Their anti-inflammatory properties, when collected from melatonin-treated MSCs, have been used to promote wound healing in diabetic skin [225]. In fact, downregulation of the proinflammatory cytokines IL-1β and TNFα as well as iNOS (inducible NO synthase) was observed, as was the upregulation of the anti-inflammatory IL-10 and the M2 macrophage marker Arginase-1. These findings strongly indicate that the exosomes promoted a change of macrophage polarization from M1 to M2, something that is otherwise known from melatonin [226]. Additionally, the upregulation of PTEN (phosphatase and tensin homolog) was reported as well as an inhibition of Akt phosphorylation [225]. This is explained by the PTEN-catalyzed dephosphorylation of PIP3 (phosphatidylinositol 3,4,5-tris-phosphate) to PIP2, a known inhibitor of Akt signaling. In a study on spinal cord injury, the melatonin-promoted M1-to-M2 shift was corroborated for both macrophages and microglia when treated with microvesicles from melatonin-preconditioned MSCs [227]. The findings concerning microglia are, again, in good agreement with corresponding actions of directly administered melatonin [59]. The microvesicles collected from melatonin-pretreated cells were also shown to be enriched with USP29 (ubiquitin-specific protease 29, systematic name: ubiquitin carboxyl-terminal hydrolase 29) [227], a deubiquitinase that prevents the proteasomal decay of Nrf2 (nuclear factor erythroid 2-related factor 2), a key transcription factor of anti-inflammatory and antioxidant signaling. This study also revealed another novel aspect of MSC function concerning the stability of *Usp29* mRNA, which was enhanced by melatonin, but strongly decreased by overexpression of METTL3 (methyltransferase-like 3), an RNA methylating enzyme, also referred to as the “writer” of adenosine 6-methylation. At first glance, these opposite effects of melatonin and METTL3 might appear to be independent, but it can be concluded that they interact or compete, since melatonin was shown to decrease m^6^A (6-methyladenosine) in overall RNA by reducing METTL3 expression [227]. This finding is worth strongly emphasizing, because it represents an entirely novel, unprecedented action mode of melatonin, which takes place at the level of regulation by RNA modification.

The anti-inflammatory properties were also used in the treatment of renal ischemia/reperfusion, in which varying combinations of melatonin, BMSCs, and BMSC-derived exosomes were compared. The combined treatment of melatonin, BMSCs, and exosomes proved to be most efficient. Again, suppression of inflammatory and oxidative markers and regulators (NF-κB) was observed and, also, reduced DNA damage (detected by comet assay) [219]. The exosomal cargos that are changed by melatonin treatment of the releasing MSCs are worth some additional considerations. First, it was shown that exosomes can also transfer small mitochondria to other cells [228]. The quantitative importance of this observation and the eventual relevance to melatonin’s actions, including mitochondrial protection and intramitochondrial melatonin synthesis, await further investigation. A further type of cargo concerns various RNAs, such as miRNAs and their sponges, lncRNAs and circRNAs, as well as mRNAs and their counterparts, asRNAs (antisense RNAs), and certainly also the various types of enhancer and super-enhancer RNAs, as summarized elsewhere [229]. To date, respective studies based on melatonin pretreatment have only considered miRNAs. Melatonin-stimulated exosomes originating from ADMSCs have recently been shown to transfer miR-34a, miR-124, and miR-135b, thereby mediating anti-inflammatory properties [230]. The many other noncoding RNAs meanwhile shown to be influenced by melatonin might motivate to conduct further studies along this line, which could open countless new technical and medical applications.

## 9. Some Aspects concerning Cancer Stem Cells

Cancer stem cells (CSCs) are a matter of particular concern as they transmit stem cell properties to their descendent tumor cells. Of course, it is of medicinal interest to explore whether melatonin may be able to reprogram CSCs with an outcome of reduced proliferation and viability. If so, it is important to identify differentiation factors that are influenced by melatonin in these cells. It is of utmost importance to be aware that findings made in nontransformed SCs cannot be translated to CSCs. Generally, the actions of melatonin are frequently opposite in nontumor and tumor cells, especially with regard to apoptosis, SIRT1 effects, and the roles of inflammatory regulators [231,232,233]. Limits of interpretation result from approaches in which CSCs are subjected to combined treatments with melatonin and a cytostatic agent. Another type of combination was applied in hepatocellular carcinoma, in which MSCs were tested for their therapeutic potential in the presence of melatonin [234]. In this case, an increase of apoptosis was reported, something that would be in accordance with experience from applications of melatonin alone in tumor cells. In breast cancer stem cells, melatonin was shown to inhibit the epithelial–mesenchymal transition [235]. Another investigation on breast CSCs reported that melatonin downregulated the expressions of the estrogen receptor Erα and of Oct4 and also reduced the binding of Erα to the *Oct4* gene [236]. Downregulation of *Oct4* by melatonin was also reported in several other studies [237,238]. In the CRC cell line MCF-7, melatonin reduced proliferation, *Oct4* and *Nanog* expression, and increased apoptosis [238]. This paper also reported upregulation of *Sox2*. The latter finding contrasts with results obtained in colon CRCs, in which melatonin downregulated *Sox2*, and also *Nanog* and *Oct4* [239]. In this study, the effects were partially attributed to a melatonin-induced suppression of PRP^c^ (cellular prion protein). However, the contribution by melatonin is not fully clear, because it was applied in combination with 5-fluorouracil. The downregulation of Oct4 via PRP^c^ suppression may be explained by a cancer-specific mode of Oct4 upregulation, in which the interacting factors PRP^c^ and c-MET (c-mesenchymal–epithelial transition), the latter being a (proto-)oncogen frequently found in tumor cells, jointly upregulate ERK1/2, which stimulates Oct4 expression [240]. With relation to osteogenic differentiation, the properties and accessibility of osteosarcoma stem cells are of interest. In these cells, melatonin was shown to downregulate Sox9, i.e., a factor that induces various osteoblast-typical genes, and to inhibit proliferation [241]. In leukemia stem cells, inhibition of proliferating self-renewal by melatonin was reported to be mediated by upregulation of miR-193a, which targets *AML1-ETO*, an oncogenic fusion gene formed by translocation, whose protein is known to increase β-catenin levels [242].

These few examples cannot provide a comprehensive picture of deviations in cancer stem cells. Moreover, the knowledge on (re-) programming influences of melatonin has still remained rather limited. Solely descriptive findings that do not allow mechanistic interpretations have been omitted. Nevertheless, the findings summarized here may already conveyed the message that continuation of work on melatonin’s actions in CSCs appears to be promising, with regard to both understanding the deviations in signaling and partial reprogramming with therapeutic outcomes.

## 10. Conclusions

The participation of melatonin in programming, maintenance of self-renewal, and proliferation of stem cells is now beyond doubt. However, it is important to distinguish between the different properties and developmental predispositions of the various types of stem cells. While some of them have retained pluripotency, others are committed to a single developmental lineage. In the latter case, especially regarding spermatogonial stem cells, megakaryocytes, and presumably pancreatic stem cells, melatonin can promote the final step of differentiation (cf. Section 3). While megakaryocytes can only decompose to platelets, other unilaterally determined stem cells are stimulated to proliferate, and, if a step of asymmetric division is involved, this contributes to both the maintenance of a self-renewing population and the expansion of the fully differentiated final cell type. In these cases, the aspect of stem cell programming is, thus, of only limited importance. However, this is different in pluripotent stem cells, in which melatonin can drive the development into different directions.

The participation of melatonin in the decision bifurcations of determination is particularly evident in NSCs and MSCs. In NSCs, melatonin seems to preferably favor neurogenesis, whereas the development to astrocytes, NG2 glia, and oligodendrocytes is tendentially suppressed, as long as neurogenesis continues (Section 6). MSCs represent a cell type with a particularly high pluripotency, which is, however, partially restricted by the tissue localization of their niches. A major decision concerning the direction of their development depends on the presence or absence of Wnt expression. Although melatonin can upregulate the important subform Wnt4 (Figure 1), it does not do this in other MSC subtypes, especially not in ADMSCs (Section 8.3). The reason for this difference is only incompletely understood. Possible explanations have focused on the microenvironment and an epigenetic memory. Nevertheless, melatonin stimulates adipogenesis in these cells by upregulating adipogenic transcription factors, such as C/EBPβ and PPAR-γ, as well as the adipocyte marker aP2. Although C/EBPβ and PPAR-γ are involved in numerous further cellular regulation processes of various cells, their high expression under the influence of melatonin is decisive for the adipogenic differentiation of ADMSCs. Notably, melatonin does the opposite in stem cells with Wnt4 expression, as it suppresses C/EBPβ, PPAR-γ, and aP2 in BMSCs. Wnt expressing stem cells can give rise to the formation of different cell types. In particular, chondrogenesis and osteogenesis have been preferentially studied. As both processes are stimulated by melatonin, the decision between them has to be made by other factors. FGFR3 has been discussed concerning this role and also the expression level of the BMP antagonist, Noggin (Section 8.4.). Regarding osteogenic differentiation, the signaling pathway from melatonin to the major osteogenic transcription factors has been widely clarified (Figure 1). These transcription factors are responsible for the expressions of various osteoblast-typical markers. The medical importance of these findings is considerable, since it offers therapeutic options in osteoporosis by reducing, in bone tissue, undesired adipogenesis in favor of osteogenesis. In this place, it should be briefly mentioned that melatonin, in addition to promoting osteogenesis, jointly suppresses the formation and activity of osteoclasts [164,243,244], effects that were even observed in osteoblast/osteoclast co-cultures [199].

Notably, melatonin can also promote the differentiation of MSCs to neural progenitor cells and further to neurons (Section 8.5). It may be important to recognize that the neurogenic development is also Wnt-dependent. Nevertheless, even ADMSCs, which do not express Wnt, have been shown to be transdifferentiated to neural progenitor cells/neurons [213]. This surprising finding has to consider the differences in an ectopic microenvironment, but also the multitude of Wnt and Frz subforms as well as the participation of ROR2. In neurogenic differentiation, Wnt5a and Frz3 seem to be decisive, and the upregulation of ROR2 indicates a contribution of noncanonical Wnt signaling [213]. Even entirely different cells such as fibroblasts have been reported to undergo neural transdifferentiation under suitable conditions [97]. These results concerning transdifferentiation offer considerable options for the treatment of neurodegenerative diseases and for the repair of CNS injuries by stroke or trauma. The advantage of the use of MSCs or other cells from non-neural lineages is clearly a matter of quantity and versatile application. Natural neurogenesis is largely confined to rather small niches in a few brain areas. Transdifferentiation of non-neural cells can be applied in many places. Moreover, transplanted stem cells can additionally promote the formation of biobridges through which endogenous precursor cells can migrate to the site of injury [147,148,149,150]. Finally, the application of melatonin can support the survival of transplanted cells by downregulating inflammatory and prooxidant processes.

Another potential field of application concerns cancer stem cells. However, it seems rather unlikely that a genetically deviating CSC that, e.g., expresses an oncogenic fusion protein or another oncogenically mutated gene could be re-programmed to normal by application of a regulatory agent such as melatonin. In these cases, the value of melatonin may be rather seen in the inhibition of proliferation, suppression of metastasis, and induction of apoptosis. 

Collectively, the actual body of evidence for actions of melatonin in stem cells has brought about a lot of new insights into signaling and programming pathways, but also revealed promising therapeutic options. 

## Figures and Tables

**Table 1:** Effects of melatonin in NSCs/NSPCs.

Cells Investigated	Challenge	Main Effects	References
NSCs from murine adult SVZ in vitro	None	Proliferation↑ differentiation of NSPCs to neurons↑	[97]
Mouse cortical NSCs	None	Proliferation↑ viability↑	[106]
Mouse cortical NSCs	None	Differentiation ↑ MT_1_ dependence, ERK activation; requirement of chromatin remodeling via H3K14 acetylation	[107]
Mouse hippocampal NSCs	None	Expression of DCX ↑	[108]
C17.2 cell line	None	Neuronal differentiation↑ MT_1_ dependence, requirement of chromatin remodeling via H3K14 acetylation	[109]
Rat midbrain NSCs	None	Viability↑ dopaminergic differentiation (tyrosine hydroxylase↑), BDNF↑ GDNF↑	[110]
Mouse NSCs from ganglionic eminence	None	Differentiation to neurons↑ in FBS-stimulated proliferation, but not in differentiation period	[111]
Murine induced pluripotent stem cells	None	Differentiation to NSCs↑ and further to neurons↑ PI3K/AKT signaling	[112]
Mouse hippocampal NSCs	None	Survival↑ differentiation↑	[113]
NSCs from adult murine SVZ	None	Proliferation↑ differentiation↑; ERK/MAPK signaling	[114,115]
Rat adult hippocampal NSCs	None	Proliferation↑ phosphorylation of ERK1/2 andc-Raf	[116]
Mouse adult spinal cord NSPCs	None	Proliferation↑ PI3K/AKT signaling	[117]
Murine hippocampus in vivo	None	Neurogenesis↑	[118]
Murine dentate gyrus in vivo	None	NeuN+ cells↑ DCX+ cells↑	[108,113,119,120,121,122,123,124]
Rat dentate gyrus in vivo	Pinealectomy	Neurogenesis↑	[125]
Rat embryonal NSCs	IL-18	Proliferation↑ differentiation↑ BDNF↑ GDNF↑	[126]
Mouse embryonic cortical NSCs	LPS	Sox2 expression↑ PI3K/Akt/Nrf2 signaling	[20]
Mouse cortical NSCs in vitro	Hypoxia	Proliferation↑ differentiation to neurons↑ MT_1_ dependence, pERK1/2↑	[19]
Mouse embryonic cortical NSCs	Hyperglycemia	Proliferation↑ self-renewal↑ autophagy↓	[127]
Murine dentate gyrus in vivo	Corticosterone	Attenuation of proliferation suppression	[128]
Rat adult hippocampal NSCs	Dexamethasone	Reversal of inhibition of nestin and Ki67 expression	[129]
Murine dentate gyrus in vivo	Dexamethasone	Attenuation of suppressed DCX expression	[130]
Murine dentate gyrus in vivo	Cuprizone	Restoration of Ki67+ proliferative cells and DCX+ NPCs; BNDF↑	[131]
Murine dentate gyrus in vivo	Scopolamine	Restoration of Ki67+ proliferative cells and DCX+ NPCs	[132]
Murine dentate gyrus in vivo	d-Galactose (aging model)	Restoration of Ki67+ proliferative cells and DCX+ NPCs	[133]
Rat adult hippocampal NSCs	Methamphetamine	Reversal of inhibition of NSC proliferation	[134]
Murine hippocampus in vivo	Methamphetamine	Nestin↑ DCX↑ β-III-tubulin↑	[135]
Rat hippocampal subgranular zone	Valproic acid	Attenuation of reduced neurogenesis	[136]
Rat hippocampal subgranular zone	Methotrexate	Attenuation of reduced neurogenesis	[137]
Rat hippocampal subgranular zone	5-Fluorouracil	Attenuation of reduced neurogenesis	[138]
Murine cortex	Ischemia/reperfusion	Nestin+ cells↑ Ki67+ cells↑ DCX+ cells↑ MT_2_ dependence	[139]
Murine cortex and striatum	Mild focal ischemia	Neurogenesis↑	[140]
Rat spinal cord	Spinal cord injury	Proliferation↑ nestin+ cells↑	[141]

Symbols: ↑ increase or upregulation; ↓ decrease or downregulation.

## Data Availability

Not applicable.
